# Improved bio-inspired with machine learning computing approach for thyroid prediction

**DOI:** 10.1038/s41598-025-03299-8

**Published:** 2025-07-02

**Authors:** Divya Kesavulu, Kannadasan R

**Affiliations:** School of Computer Science and Engineering, Vellore Institute of Technology (VIT), Vellore, 632014 India

**Keywords:** Thyroid, Machine learning, Deep learning, Optimisation techniques, Feature selection, Particle snake swarm optimization, RF, DT, SVM, KNN, CNN-LSTM, Health care, Engineering

## Abstract

Thyroid illness is widely recognised as a prevalent health condition that can result in a range of health disorders. Thyroid illnesses, namely hypothyroidism and hyperthyroidism, are widespread worldwide and present considerable health consequences. These conditions have a particularly noticeable effect on women in places such as Asia, Latin America, and Africa. Precise and prompt identification of thyroid diseases is essential because of their significant impact on metabolism and general well-being. This study investigates the use of several machine learning (ML) and deep learning (DL) methods, such as random forest (RF), decision tree, SVM, and KNN, to improve the precision of predicting thyroid illnesses. The objective is to enhance the performance of these models by using more sophisticated optimization methods like particle snake swarm optimization (PSSO). The results of the analysis, assessed using parameters such as accuracy, recall, precision, F1-score, and Specificity, clearly show substantial improvements in predictive capability. The random forest with PSSO model attained an accuracy of 98.7%, an F1-score of 98.47%, a Precision of 98.51%, a Recall of 98.7%, and a Specificity of 98%. Notably, PSSO-RF outperformed a CNN-LSTM deep learning baseline by 2.98% (95.72%) in accuracy, highlighting the effectiveness of bio-inspired optimisation in improving conventional machine learning models. This research adds to the increasing data of research that uses computational innovations to improve healthcare outcomes. The results indicate that by using RF with PSSO optimiser, an accuracy score of 98.7% can be achieved. This performance surpasses that of current state-of-the-art models.

## Introduction

According to WHO (the World Health Organization), thyroid disease is the second most prevalent endocrine gland problem globally, behind diabetes. Hypothyroidism and hyperthyroidism are the predominant thyroid gland disorders, documented in over 110 countries worldwide, posing a risk to 1.6 billion individuals^1^. Most affected individuals reside in Asia, Africa, and Latin America.

Thyroid diseases affect almost 42 million individuals in India, with women being disproportionately impacted. The incidence of thyroid problems in women is significantly greater than in males, as indicated by the female-to-male ratio^1^. Hypothyroidism has a significant impact on many women. Approximately 32 million individuals in the nation are believed to have hypothyroidism, with a substantial majority of them being women^2^. The disorder is more common in older age groups, particularly in women aged 35 and beyond. The thyroid gland governs several physiological processes in the front region of the neck. Regrettably, there has been a noticeable originate in the incidence of thyroid disorders, predominantly among the female population, in recent years^3^.

Thyroid illness is a prevalent medical ailment that affects the thyroid, an organ shaped like a butterfly located in the neck. The thyroid is responsible for releasing hormones that regulate metabolism^4^. The condition may be classified into 4 distinct forms: thyroiditis, Hypothyroidism, hyperthyroidism, and Hashimoto’s thyroiditis. It is characterised by either an excessive or inadequate synthesis of hormones^5^. Detecting thyroid illness is a challenging and complex job that requires much knowledge and data. Thyroid illness identification has become an essential skill due to recent advancements in medical information systems. This is because thyroid dysfunction exerts a substantial impact on other organs inside the body^6^. The thyroid gland’s synthesis of thyroid hormones facilitates the control of several physiological processes, such as heart rate and body temperature, that are associated with the body’s metabolic rate. The thyroid gland yields 2 types of hormones: triiodothyronine (T3) and levothyroxine (T4)^7^. These hormones are fundamental for the process of protein synthesis, the regulation of body temperature, and the creation and control of total energy. Thyroid problems should not be underestimated, since thyroid storm (an intense period of hyperthyroidism) and Myxedema coma (the end phase of untreated hypothyroidism) can be deadly in a significant number of instances^8^. An estimated proportion of mature women, ranging from 9 to 15 percent (15%), and a smaller number of males are believed to be affected. Thyroid illness may be broadly categorized into two main groups: those that affect the normal working of the thyroid secretor, and those that involve the development of thyroid tumours or neoplasms. Most thyroid problems can be effectively addressed^9^. Thyroid dysfunction commonly arises from either inadequate production or overproduction of thyroid hormone. Hypothyroidism is the medical word for the inadequate production of thyroid hormone, whereas hyperthyroidism is the term used to describe the excessive production of thyroid hormone^10^. Thyroid dysfunction is a prevalent condition that affects individuals of all age groups. While the disease itself may not pose a significant threat compared to conditions such as heart disease and cancer, it has the potential to lead to other diseases that can have serious repercussions. Timely and accurate diagnosis is significant for the management and treatment of thyroid disorders^11^. Traditional diagnostic methods mostly rely on biochemical analyses and clinical evaluations, which, although effective, can be labour-intensive and resource-demanding^12^.

Conventional diagnostic methods for thyroid disease depend on biochemical tests and clinical evaluations, which are laborious, costly, and require laboratory equipment. Misinterpretation of data or delayed diagnosis may lead to inappropriate treatment alternatives^13^. This has underscored the necessity for automated, data-driven methodologies to enhance the detection and risk assessment of thyroid disorders. Machine learning (ML) and deep learning (DL) have become key technologies in healthcare and medical diagnostics, providing solutions that enhance accuracy, efficiency, and early disease detection^14^.

The advent of ML and DL has transformed thyroid disease prediction, offering automated and accurate diagnostic tools. Traditional diagnostic methods, including biochemical tests (TSH, T3, T4) and imaging techniques, are often slow, costly, and susceptible to human mistakes^1^. Several ML algorithms, such as decision trees (DT), RF, support vector machines (SVM), and K-Nearest Neighbors (KNN), are frequently employed to examine structured numerical data, including patient demographics and lab results, enabling early detection and minimizing misdiagnosis^15^. Conversely, DL approaches, like artificial neural networks (ANNs), long short-term memory (LSTM) networks^16^, and Autoencoders, can identify complex relationships in thyroid-related information without explicit feature selection. While ML models are computationally efficient and effective for structured datasets, DL models excel at identifying intricate patterns and sequential trends, making them particularly useful for analyzing thyroid function over time^17^.

Nevertheless, the precision and efficiency of these models rely on the selection of features and hyperparameters. Conventional methods for optimizing hyperparameters, such as grid search and random search, can be computationally intensive and time-consuming. To tackle this issue, bio-inspired optimization techniques, including particle swarm optimization (PSO) and genetic algorithms (GA), have been widely utilized to enhance feature selection, hyperparameter tuning, and overall model accuracy^18^. More recently, advanced hybrid techniques, such as snake optimization algorithm (SOA) and particle snake swarm optimization (PSSO), have demonstrated potential in further improving classification performance and reducing computational expenses. This research investigates the combination of machine learning models (DT, RF, SVM, KNN) with optimization techniques (PSSO) to enhance the accuracy of thyroid disease prediction. By fine-tuning model parameters, this approach aims to deliver more dependable, efficient, and automated healthcare solutions, not only for thyroid disorders but also for wider applications in medical diagnostics.

### Motivation and context

This study is motivated by the rising need for reliable computational tools in healthcare to solve the challenges of traditional thyroid diagnostic approaches. Machine learning and deep learning have emerged as useful methods for analysing large datasets and identifying patterns that manual diagnosis may overlook. Nonetheless, optimal predictive performance is dependent on good feature selection and hyperparameter tuning to prevent overfitting and ensure model generalisation.

Conventional approaches, which rely on biochemical testing (TSH, T3, T4) and clinical evaluations, are lengthy, costly, and need laboratory facilities. These procedures are prone to human error and delayed diagnosis, which might result in improper therapy and consequences. Automated, data-driven solutions provide faster and more accurate diagnostics, reducing reliance on manual interpretation and cutting diagnostic times. Nonetheless, achieving high prediction accuracy necessitates careful feature selection and hyperparameter tweaking. Ineffective feature selection can result in noise, and inadequate hyperparameter settings might induce overfitting or weak generalisation, jeopardising prediction model reliability.

The primary research objective of this study is to select the features and achieve high accuracy in predicting thyroid illness. Given the study topic that has been mentioned, we can formulate many research questions:

(i) How can we determine the optimal number of features in a medical dataset while still achieving high classification accuracy?
(ii) Which search method is most appropriate for managing the FS process?
(iii) Which improvement operators may be used with the search algorithm to improve its performance?
(iv) Which assessment metrics should be used to assess the performance of the created algorithm?

To address these difficulties, this research investigates the integration of Bio-inspired optimisation approaches, specifically PSSO, with machine learning models to improve feature selection and parameter tuning. This technique intends to improve prediction accuracy and computing efficiency, therefore contributing to the creation of reliable, automated diagnostic tools for thyroid illness and other medical applications.

The objective of this exploration is to progress a modelling solution for predicting thyroid illness, to allow society to reap the benefits of computational developments in this field. We have utilised feature selection methodologies, and the resulting output from these methodologies is fed into classifiers. The comparison study demonstrates the effectiveness of our strategy. To create a dependable prediction method, it is crucial to meticulously opt for the most applicable ideal pair of parameters and machine learning algorithms. The suggested model’s outcomes are estimated using several execution measures, including accuracy, F1-score, recall, and precision.

The primary aims of the modern research endeavour are as follows:

- Data preprocessing- Firstly, the thyroid dataset is obtained from the dataset and the redundant, missing data and noise in the input dataset are eliminated.
- Following pre-processing, the balanced dataset is sent to the optimized machine learning classifiers to predict thyroid illness.
- To improve the performance of the ML classifiers, the features are carefully chosen using a hybrid particle snake swarm optimization approach (PSSO). The suggested PSSO is a combination of particle swarm optimization (PSO) and snake swarm optimization (SSO).
- The suggested approach’s performance is evaluated using several metrics and compared to state-of-the-art works to determine its effectiveness.

The subsequent sections of this document are structured in the following manner: Section “Literature work” discusses the literature work; Section “Materials and methods” discusses the proposed model in detail; Section “Results” discusses the results and discussion, of computational performance metrics, Section “Discussion” discusses and future directions, and section “Conclusion” conclusion.

## Literature work

Deep and machine-learning approaches have been used in previous studies to predict thyroid illness. Early detection and classification of thyroid illness are crucial for optimal treatment and recovery for patients. This study presents contemporary research on thyroid illness categorisation and detection methods.

This study^19^ employed machine learning algorithms, including random forest, gradient boosting, logistic regression, support vector machines (SVM), and deep neural networks (DNN), to predict molecules likely to disrupt thyroid hormone homeostasis with high accuracy. Early prediction of such molecules is crucial for prioritizing further experimental testing in the early phases of thyroid disease research. The biological data used in these experiments were derived from the ToxCast dataset. According to the article, the predictive performance was highest for thyroid peroxidase (TPO) and Thyroid Hormone Receptor (TR), with F1 scores of 0.83 and 0.81, respectively, indicating strong model efficacy in identifying key molecular changes.

This study^20^ employed a machine learning framework for early-stage thyroid illness prediction utilising several classification approaches, including SVM, DT, RF, LR, and NB. The researchers used the UCI Machine Learning Repository dataset, which has 519 instances and 15 properties, including important features like TSH, T3, and TT4. Data preparation approaches, such as noise reduction and managing missing values, were used to improve model performance. According to the study, the Random Forest classifier outperformed the other models with an accuracy of 97.05%. Furthermore, Recursive Feature Elimination (RFE) was employed for feature selection, which considerably improved prediction accuracy. The work emphasises the importance of early hypothyroidism identification and proposes further research using larger datasets and dimensionality reduction approaches to improve diagnostic speed and efficiency.

This study^21^ examines a model for predicting thyroid disease using butterfly optimized feature selection with fuzzy C-means classifier (DEBOA-FCM) to improve diagnostic precision. The researchers seek to minimize feature redundancy and enhance feature selection by combining differential evolution (DE) and butterfly optimization algorithm (BOA). The fuzzy C-means (FCM) algorithm is employed for classification, categorizing thyroid conditions into normal, hypothyroid, and hyperthyroid. The research utilizes data from the UCI machine learning repository, encompassing 4152 instances and 18 features. The proposed method was compared against individual algorithms, achieving 94.3% accuracy, which significantly surpassed differential evolution (88.4%), butterfly optimization algorithm (90.6%), and fuzzy C-means (89.9%). Additionally, DEBOA-FCM demonstrated high precision (89.78%), recall (93.45%), and F1-score (91.44%), while reducing computational time compared to alternative methods. The findings indicate that the DEBOA-FCM approach effectively optimizes feature selection while minimizing errors and computational expenses.

This study^22^ examined a model for predicting thyroid illness that combines ant lion optimisation (ALO) and long short-term memory (LSTM) to increase prediction accuracy while reducing computational complexity. The researchers tackle the high-dimensionality challenge by applying entropy-based feature selection to reduce the feature set from 31 to 10, with an emphasis on essential characteristics like TSH, T3, TT4, and FTI. The UCI machine learning repository dataset, which contains 9,172 instances and 31 characteristics, was utilised for training and assessment. The ALO method was used to optimise LSTM hyperparameters such as learning rate, batch size, and neurone count, resulting in faster convergence and greater accuracy. The suggested ALO-LSTM model outperformed the baseline LSTM model with the Adam optimiser, achieving 98.6% accuracy versus 91.4%.

This study^23^ examines a unique strategy for identifying thyroid illnesses that combines the synthetic minority oversampling technique for nominal and continuous features (SMOTE-NC) with a fine-tuned light gradient boosting machine (LGBM) model. The study looks at the problem of class imbalance in a thyroid illness dataset with 3,772 patient records and compares the performance of various machine learning and deep learning models. The SMOTE-NC-LGBM (SNL) strategy improves classification accuracy by 96%, exceeding existing machine learning and deep learning techniques.

This study^24^ introduces a quantum computing-based machine learning framework for thyroid illness prediction that uses quantum particle swarm optimisation (QPSO) for feature selection and quantum support vector machine (QSVM) for classification. When tested on a dataset of 7200 instances, the model scored 98.77% accuracy, 99% precision, recall, and F1-score, surpassing Random Forest, KNN, and traditional SVM. The quantum-based technique enhances computing efficiency and accuracy, making it a potentially useful tool for real-time clinical diagnostics.

This study^25^ introduces a deep learning-based model for identifying and categorising five different forms of thyroid disease: hypothyroidism, hyperthyroidism, thyroid cancer, thyroiditis, and thyroid nodules. Using a modified ResNet architecture with convolutional neural networks (CNN), the model improves performance by applying dual optimizers—Adam and stochastic gradient descent (SGD)—during training, attaining an accuracy of 97%, up from 94% with the standard ResNet design. Preprocessing procedures guarantee that images are the same size and quality, and the model’s performance is tested using measures like accuracy, recall, and F1 score. A web-based platform built with ReactJS supports real-time categorisation, allowing users to input thyroid photos for fast diagnosis. The suggested model outperforms previous CNN-based architectures including VGG16, VGG19, and InceptionV3 in terms of accuracy and precision, making it an effective option for thyroid disease detection.

The study^26^ exhibits a hybrid model that combines the Deep Convolutional Neural Network (DeepCNN) for classifying thyroid diseases with the adaptive tunicate swarm algorithm (ATSA) for feature selection. To increase accuracy, the DeepCNN’s hyperparameters are tuned using the grey wolf optimiser (GWO). By employing GWO to optimise the DeepCNN structure and choose the best features, the study seeks to improve classification performance.

The study^16^ exhibits a hybrid deep-learning strategy for detecting and classifying thyroid illness based on ultrasound pictures. The fundamental approach consists of integrating Vgg-19 for feature extraction and LSTM for classification. To enhance feature selection and hyperparameter tweaking, a hybrid meta-heuristic optimisation approach called black widow optimisation (BWO) and mayfly optimisation algorithm (MFO) is utilised. The DDTI dataset, which contains 99 patients and 134 thyroid ultrasound pictures, is used for assessment. The suggested technique outperforms previous models such as CNN, ResNet-50, and DNN in terms of accuracy and computing efficiency.

This research^27^ leverages machine learning to diagnose thyroid illness early and accurately, overcoming shortcomings in standard biochemical diagnostics. A dataset of 9,173 patient records was analysed using a Random Forest ensemble model, as well as LR, SVMs, and DT, to incorporate significant clinical and biochemical variables. Model performance was improved by using feature selection and dimensionality reduction techniques like PCA and RFE. The RF classifier obtained 92.2% accuracy, accurately recognising 1844 true positive instances with a 90.3% recall rate and an F1-score of 0.90, indicating its efficacy. The findings emphasise the potential of machine learning (ML)-driven models to improve early diagnosis, minimise delays, and integrate into clinical processes for better patient care and healthcare efficiency.

The examined research explores diverse machine learning and deep learning algorithms for thyroid illness diagnosis including drawbacks, such as limited datasets, unbalanced datasets, and inadequate emphasis on model optimisation. These gaps limit generalisability, increase the risk of bias and produce false positives. The study’s goal is to overcome these challenges by focusing on hyperparameter optimisation with the PSSO method, emphasising the necessity of fine-tuning for accurate predictions. A comparative analysis of cutting-edge methodologies is presented in (Table 1).

**Table 1:** A Summary of the systematic analytical research studies in related work.

Study	Year	Dataset	Methods	Results	Drawback
^19^	2021	ToxCast	Random forest, gradient boosting, logistic regression, SVM, DNN	F1-score-83%	The model’s dependence on the ToxCast dataset restricts its generalizability to broader chemical spaces and lacks model Interpretability
^20^	2022	UCI	SVM, Decision tree, RF, LR, Naïve Bayes	Accuracy- 97.05%	The dataset has only 519 samples, which may result in overfitting and decreases the model’s generalisability. It just reports accuracy and does not provide vital metrics
^21^	2023	UCI	Differential Evaluation, Butterfly Optimization Algorithm (BOA), FCM	Accuracy-94.3%, Precision-89.78%, Recall-93.45%, F1 score-91.4%	DEBOA-FCM reduces errors and optimize features, but it has flaws such as high computational costs, restricted generalizability, overfitting risk, and no real-time validation
^22^	2024	UCI	Ant-lion optimization (ALO), LSTM	Accuracy-98.6%, Precision-99.2%, Recall-89.6%, F1 score-98.6%	Class imbalances are not handled properly, resulting in biassed forecasts and poor dependability for minority classes
^23^	2024	UCI	SMOTE-NC, logistic regression, SVM, RF, LightGBM, GRU, LSTM	Accuracy- 96%	The SMOTE-NC-LGBM model has high computational cost due to oversampling and hyperparameter tuning. Additionally, SMOTE-NC may cause to overfitting, as synthetic samples might not accurately reflect real-world data
^24^	2023	UCI	RF, KNN, SVM, QSVM	Accuracy-98.7%, Precision-99%, Recall-99%, F1 score-99%	The suggested model has a high computational cost due to the use of quantum-based approaches such as QPSO and QSVM
^25^	2023	UCI	CNN-ResNet, VGG16, VGG19, InceptionV3, Adam, SGD	Accuracy-97%, Precision-98%, Recall-96%, F1 score-97%	The dataset size is small and from a few sources, which may limit its accuracy in real-world applications
^26^	2023	UCI	ATSO-DeepCNN-GWO	Accuracy-92%, F1-score-94%, Precision-95%, Specificity-95%	The significant use of optimisation techniques (GWO and ATSA) may result in overfitting, particularly if adequate cross-validation measures are not explicitly stated
^16^	2023	DDTI (image dataset)	BWO-MFO, Vgg-19-LSTM	Accuracy-98.8%, Precision-99.2%, Recall-98.6%, F1 score-98.9% Specificity-89.16%	The model’s small dataset raises the possibility of overfitting, and its high computing complexity leads to it being inappropriate for real-time clinical applications
^27^	2025	Kaggle	RF, DT, SVM, LR, PCA, RFE	Accuracy- 92.2%, Recall-90.3%, F1-score- 90%	This study faces computational complexity difficulties, making real-time applications difficult. Moreover. The dataset’s minimal variety may restrict its applicability across diverse groups

Table 2 provides a summary and comparison of recent ML, DL, and Hybrid models utilised in the prediction of thyroid disease. Conventional ML models demonstrate effectiveness with small datasets, whereas DL models utilise image datasets but encounter constraints related to dataset size. Hybrid models that integrate ML and DL with optimisation techniques, including ALO, ATSA, and QPSO, exhibit enhanced accuracy and scalability. The proposed study demonstrates superior results compared to previous studies’ research by utilising PSSO combined with classical ML classifiers on an extensive dataset with 22,632 samples. It achieves an accuracy of 98.70% while effectively addressing class imbalance through the application of SMOTEENN. Hybrid models demonstrate enhanced performance, especially in the context of high-dimensional clinical data.

**Table 2:** Taxonomy for ML vs. DL vs. Hybrid models.

Study	Approach type	Algorithms used	Dataset size	Accuracy	Feature selection/optimisation
^20^	ML	RF, SVM, DT, NB, LR	519 (UCI)	97.05	RFE
^25^	DL	CNN-ResNet, VGG16, VGG19, InceptionV3	1200 (UCI)	97	Adam, SGD
^22^	Hybrid (DL + Opt)	ALO-LSTM	9172 (UCI)	98.6	Entropy based FS + ALO
^26^	Hybrid (DL + Opt)	DeepCNN + ATSA + GWO	7200 (UCI)	92	GWO + ATSA
^21^	Hybrid (ML + Opt)	DEBOA + FCM	4152 (UCI)	94.3	DE + BOA
^24^	Hybrid (Quantum ML)	QSVM + QPSO	7200 (UCI)	98.77	QPSO
This Study	Hybrid (ML + Opt)	PSSO- RF, PSSO-DT, PSSO-SVM, PSSO-KNN	22,632 (Figshare)	98.70	PSSO + SMOTEENN

## Materials and methods

**Fig. 1:**
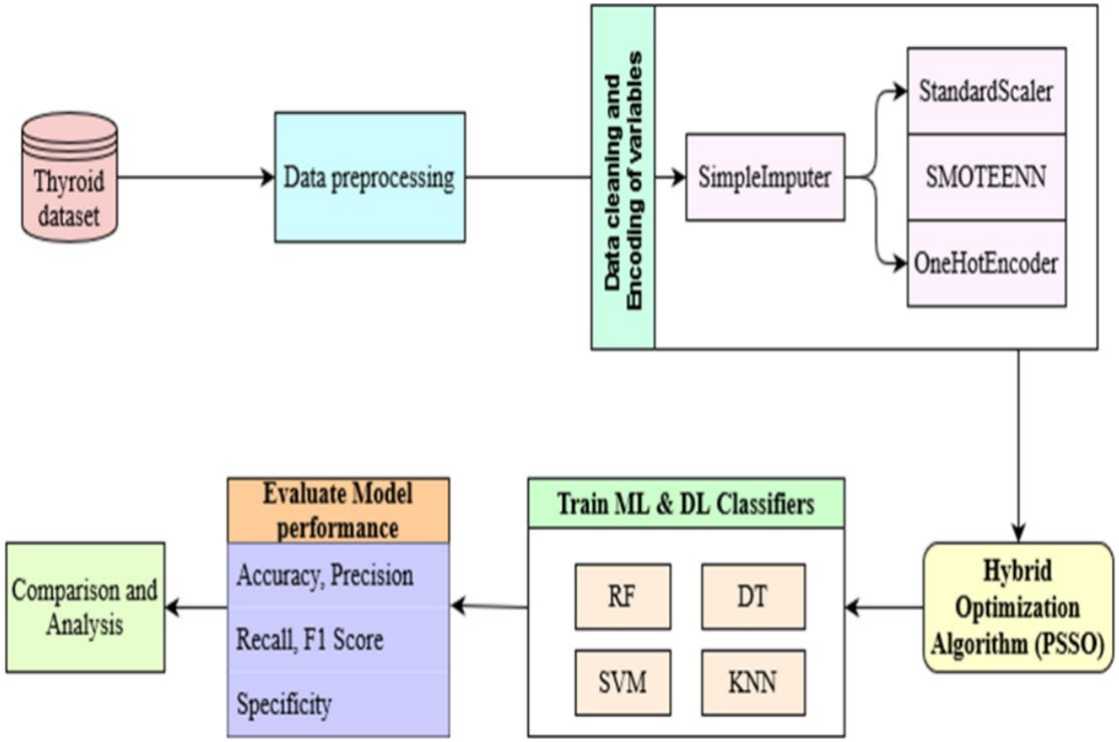
Block diagram of the proposed methodology.

This paper proposes a machine-learning methodology for predicting thyroid disease. The study’s approach is illustrated in (Fig. 1). The initial and essential phase in the creation of a system Machine-learning model is the gathering of data. The records were obtained from the UCI machine learning repository. The dataset on thyroid disorders consists of 22632 samples, each including 31 distinct attributes. The collection has different thyroid illnesses and their corresponding target classes, out of which only five target classes are chosen for the trials. The selection of these classes is based on the abundance of samples. Due to the incomplete number of samples, the other classes were excluded from this study. To tackle the issue of inequality in the selected dataset, we employed the SMOTEENN approach, which combines the synthetic minority oversampling TechniquE with edited nearest neighbours. This approach involves the integration of over-sampling the minority set using the Synthetic Minority Over-sampling TechniquE (SMOTE) and the cleaning of the dataset using the edited nearest neighbour (ENN) algorithm. The SimpleImputer is used to replace all the records with faulty values with optimum values. Data splitting entails the partitioning of the dataset into 2 distinct sets: a training set and a testing set. The ratio employed is 0.8 to 0.2, indicating that 80% of the records are assigned for training and 20% are assigned for testing. The StandardScaler technique is utilised to standardise each feature by transforming them to ensure a mean of 0 and a standard deviation of 1, so normalising all the characteristics to the suitable coefficient. Subsequently, the OneHotEncoder is used to convert every categorical column into a binary encoding, wherein each category is denoted by either a 0 or a 1. The column transformation is utilised to ensure that both numerical and categorical transformations are applied utilising the Column Transformer. This feature enables the use of distinct preparation procedures to individual columns within a same dataset. We utilise a training set to train Machine learning models and employ the PSSO optimiser to do hyperparameter optimization. This optimizer assists in selecting the optimal hyperparameter structure for the models. Ultimately, we assess models based on their accuracy, recall, precision, F1 score, and specificity.

**Fig. 2:**
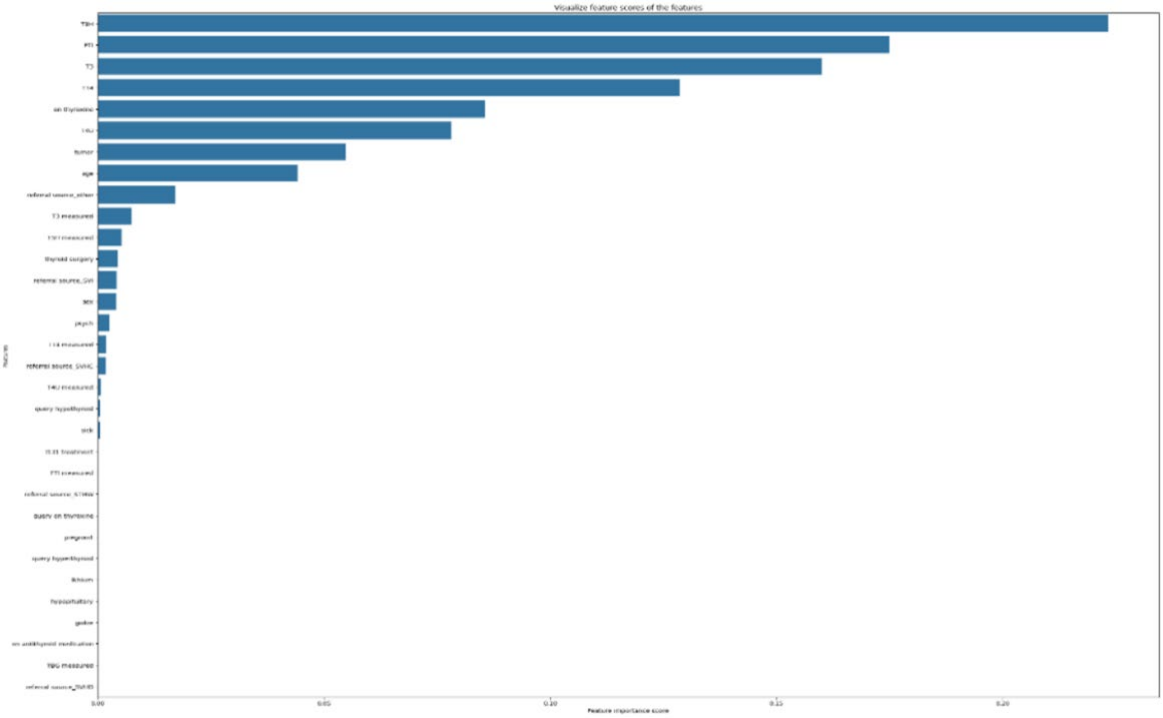
PSSO model features importance from the dataset.

After training the PSSO model on the pre-processed data, we identified the non-zero values as the most significant characteristics for predicting the target parameter. These crucial characteristics were subsequently utilised as input for the ultimate Machine learning model, which was trained and assessed using conventional procedures. Figure 2 illustrates the significant features of the thyroid dataset, respectively. Figure 2 outlines the significance of the characteristics in the PSSO model as derived from the dataset.

### Thyroid disease dataset

This repository^28^ has data on 22,632 people, including 31 attributes about thyroid diseases. Notable characteristics comprise:

- Population characteristics: patient_id, age, gender.
- Medical records: thyroid surgery, on thyroxine, I131 treatment, on antithyroid medication, pregnant.
- Laboratory metrics: TSH, TT4, T3, FTI.
- Classification: class, referral source.

The target variable, also known as the class variable, distinguishes between different types of thyroid diseases. The dataset furthermore has a variable called dataset_name, which specifies the origin or category of the data.

### Data pre-processing

Before any data analysis, it is essential to start by preparing the datasets. The data pre-processing procedure started by identifying any missing values^29^. Our dataset contains missing values, which are addressed by either replacing them with the average value of the feature in the database or removing the whole record if there are several missing values for it^30^. Another concern arises when it comes to normalising data points since certain properties exhibit distinct ranges of values. Normalisation is necessary to mitigate the disproportionate influence of one property on other qualities^30^.

### Data balancing using SMOTEENN

SMOTEENN is a potent hybrid strategy used to tackle class imbalance in datasets, making it especially valuable for datasets like the thyroid dataset where some classes may be inadequately represented. The technique integrates two separate methodologies: SMOTE, which concentrates on producing artificial samples to enhance the representation of under-represented classes, and ENN, which purges the dataset by eliminating noisy or borderline samples from both majority and minority classes. By using this dual method, it is possible to generate a dataset that is both well-balanced and free of impurities, resulting in improved performance of ML models. The SMOTE algorithm creates additional data points by interpolating between current samples and their closest neighbours within the minority class. This ensures that the newly generated samples are significant and not just replicas. Subsequently, ENN examines the vicinity of each sample and eliminates those that do not exhibit strong alignment with the bulk of their neighbouring samples, therefore successfully decreasing noise. When used on the thyroid dataset, SMOTEENN reduces the imbalance towards the majority class, allowing the model to learn more efficiently from all classes and eventually enhancing its ability to identify under-represented thyroid diseases. Implementing this approach is straightforward using Python modules like imblearn, providing a realistic option for addressing class imbalance problems in medical datasets.

### Snake optimizer (SO)

The snake optimizer (SO) algorithm, introduced by Hashim et al., is a novel optimization model that draws stimulation from the innate behaviour of snakes, such as eating, mating, and fighting. SO categorises the population into males and females and simulates their behaviour by considering ambient elements such as temperature, which influences their activities^31^. The method consists of two primary stages: exploration, during which snakes actively seek solutions in situations with little data and difficult settings, and exploitation, which entails a concentrated local search for the most optimum solutions^32^. Simulated annealing (SA) starts with randomly generated solutions and is recognised for its rapid convergence, resilience, powerful search capabilities, and little complexity, rendering it appropriate for tasks with restricted computational resources^33^. Despite facing typical obstacles such as local minima and unbalanced exploration and exploitation, several improvements have been suggested to better the performance of SO^34^.

The below points illustrate the primary phases of SO: the search process initiates by generating a collection of random responses^33^ in the area of search space using Eq. (1).

1 $${Snake}_{i}={Snake}_{min}+rand\times \left({Snake}_{max}-{Snake}_{min}\right)$$

*Snake*_*i*_ represents the point of the *i*^th^ solution in the swarm’s search space. The variable rand represents a random integer that belongs to the interval [0,1]. The lowest and maximum values for the examined issues are *Snake*_*max*_ and *Snake*_*min*,_ respectively^33^.

Snake population segmentation:

The population is evenly divided into two distinct categories: men and females.

2 $${N}_{male}=\frac{N}{2}$$

N represents the whole snake population. The population on male snakes, denoted as N_male_, is equal to half of the overall snake population^31^.

3 $${N}_{female}\approx N-{N}_{male}$$

The number of female snakes, denoted as N_female_, may be estimated by subtracting the number of male snakes denoted as N_male_, from the entire population of snakes.

Obtain the optimal answer from the female group (Sbest_female_) and the male group (Sbest_male_) and determine the whereabouts of the food L_food_. Two more notions are defined, namely the amount of food (Q), and the temperature (Temp), as represented by Eqs. (4) and (5) respectively^31^.

4 $$Q={K}_{1}\times exp(\frac{C-T}{T})$$

5 $$Temp={exp}^{(\frac{-C}{T})}$$

Let K1 be a constant value of 0.5. let C represent the current epoch and T represent the total number of epochs.

Food is sought within the search region, and snakes reposition themselves to a randomly selected location when the threshold value Q is set to 0.25. This repositioning is done using mathematical eqs. (6)–(9) designed for global search^35^.

6 $${S}_{malei}\left(C+1\right)={S}_{maleR}(C)\pm K2\times {AB}_{male}\times (({S}_{max}-{S}_{min})\times R+{S}_{min})$$

Let R represent a arbitrary number between 0 and 1. S_malei_ signifies the position of the i^th^ male snake, whereas S_maleR_ represents the location of a randomly selected Male snake. The skill of the male snake to discover food is denoted as AB_male_ and may^36^ be determined using Eq. (7).

7 $${AB}_{male}={exp}^{(-{Fitness}_{maleR}/{Fitness}_{malei})}$$

K2 represents a constant value of 0.05. *Fitness*_*maleR*_ refers to the aptness of the *S*_*maleR*_ snake, whereas *Fitness*_*malei*_ represents the ability of the *i*^*th*^ snake in the male group^36^.

8 $${S}_{femalei}\left(C+1\right)={S}_{femaleR}\pm K2\times {AB}_{female}\times (\left({S}_{max}- {S}_{min}\right)\times R+{S}_{min})$$

R represents a randomly generated number between 0 and 1. *S*_*femalei*_ refers to the location of the *i*^*th*^ Female snake. *S*_*femaleR*_ represents the perception of a randomly selected Female snake. *AB*_*female*_ denotes the competence of the Female snake to find food^37^.

9 $${AB}_{female}={exp}^{({-Fitness}_{femaleR}/{Fitness}_{femalei})}$$

The fitness values of a particular female and male snake in their respective groups are denoted as *Fitness*_*maleR*_ and *Fitness*_*female*_*. Fitness*_*femalei*_* and fitness*_*malei*_ denote the level of fitness of the *i*^*th*^ solution in the Female and Male groups, respectively^38^. The constant value K2 is precisely defined as 0.05.

Utilising the seek area (food is found): The warmth is assessed when the quantity of nutrition exceeds a specified level of Q > 0.25. The resolutions^31^ will only migrate towards the food if the temperature exceeds 0.6 (hot).

10 $${S}_{\left(i,j\right)}\left(C+1\right)={L}_{food}\pm K3\times Temp\times R\times ({L}_{food}-{S}_{\left(i,j\right)}\left(C\right)$$

The variable *S*_*(i,j)*_ denotes the position of a snake, regardless of its gender. *L*_*food*_ signifies the top-performing snakes, whereas K3 is a persistent number set to 2. If the Temp is more than 0.6 (chilly condition), the snake will enter either the contest mode or the mating stage^33^.

11 $${S}_{malei}\left(C+1\right)={S}_{malei}(C)\pm K3\times {FA}_{male}\times R\times ({S}_{femalebest}-{S}_{malei}\left(C\right))$$

The variable *S*_*malei*_ represents the position of the *i*^*th*^_male,_ *S*_*femalebest*_ represents the location of the best snake in the Female group, and *FA*_*male*_ represents the fighting capacity of the male snake^34^.

12 $${S}_{femalei}\left(C+1\right)={S}_{femalei}(C)\pm K3\times {FA}_{female}\times R\times ({S}_{malebest}-{S}_{femalei}(C)$$

Here, *S*_*femalei*_ represents the *i*^*th*^ female position, *S*_*malebest*_ represents the top male snake, and *FA*_*female*_ represents the female snake’s fighting skill. *FA*_*male*_ and *FA*_*female*_ can be determined using these equations^38,39^:

13 $${ FA}_{male}={exp}^{({-Fitness}_{femalebest}/{Fitness}_{i})}$$

14 $${FA}_{female}={exp}^{({-Fitness}_{malebest}/{Fitness}_{i})}$$

The term *Fitness*_*malebest*_ represents the highest level of physical fitness among male snakes. *Fitness*_*i*_ refers to the fitness level of the *i*^*th*^ snake, and *Fitness*_*femalebest*_ represents the highest level of physical fitness among female snakes^39^.

Mating mode:

15 $${S}_{malei}\left(C+1\right)={S}_{malei}(C)\pm K3\times {MA}_{male}\times R\times (Q\times {S}_{femalei}\left(C\right)-{S}_{malei}\left(C\right))$$

16 $${S}_{femalei}\left(C+1\right)={S}_{femalei}\left(C\right)\pm K3\times {MA}_{female}\times R\times \left(Q\times {S}_{malei}\left(C\right)-{S}_{femalei}\left(C\right)\right)$$

The locations of the *i*^*th*^ snake in the Female and Male groups are denoted as *S*_*femalei*_ and *S*_*malei*_, respectively. The role of males and females for breeding is represented as *MA*_*male*_ and *MA*_*female*_, respectively. These values can be obtained by the following methods or discovered through the following procedures^39^:

17 $${MA}_{male}={exp}^{({-Fitness}_{femalei}/{Fitness}_{malei})}$$

18 $${MA}_{female}={exp}^{({-Fitness}_{malei}/{Fitness}_{femalei})}$$

If the reproductive cell successfully emerges, select the most undesirable female and male snakes and replace them.

19 $${S}_{maleworst}={S}_{min}+R\times ({S}_{max}-{S}_{min})$$

20 $${S}_{femaleworst}= {S}_{min}+R\times ({S}_{max}-{S}_{min})$$

Among the male group, the most unfavourable snake is *S*_*maleworst*_, but in the female group, the most unfavourable solution is *S*_*femaleworst.*_ The diversity factor operator ± allows for the manipulation of snake placements in the search area, enabling the ability to adjust their positions in any direction^31^.

### Particle swarm optimization (PSO)

The PSO algorithm, initially proposed by Eberhart and Kennedy in 1995 and further refined by Eberhart and Shi in 1998, draws inspiration from the social behaviours shown by animals such as fish, birds, and insects, as well as the ideas of swarm intelligence^40^. This method emulates the combined actions of a group of individuals to address optimization challenges. Inside the context of PSO, the swarm is comprised of a group of particles, with each particle, indicating a promising solution inside the search space. In the beginning, every particle is given an arbitrary location and motion within the area being searched. Each particle aims to get the best possible answer by continuously modifying its position using its own past experiences and the experiences of other particles^41^.

Each particle’s mobility is governed by many factors:

- Velocity: The particle’s current momentum facilitates its probe of the search space.
- Personal best position: The optimal position that the particle has achieved thus far, which serves as a guiding factor for its future movements based on its own past performance^42^.
- The global best location refers to the most optimal position discovered by any particle in the swarm. It served as a directing point for all other particles, depending on the overall success achieved by the whole swarm^43^.

The formula used to update the velocity of each particle is as follows:

21 $${V}_{id}={V}_{id}\times w+C1\times rand()\times \left({P}_{id}-{X}_{id}\right)+C2\times rand()\times ({P}_{gbest}-{X}_{id})$$

Here, the symbol *V*_*id*_ signifies the particle *i*’s velocity in dimension *d.* w represents the motion weightiness, which helps balance the particle’s ability to explore and exploit^44^. C1 and C2 are velocity coefficients that indicate the particle’s inclination to approach its individual best position *P*_*id*_ and the overall best position *P*_*gbest*_, accordingly. The function *rand()* generates a random number varying from 0 to 1, so introducing a stochastic component to the movement^44^. Subsequently, the position of each particle is reorganized with the following formula:

22 $${X}_{id}={X}_{id}+{V}_{id}$$

*X*_*id*_ denotes the present location of the particle.

The PSO method operates in an iterative manner, where each particle updates its velocity and location until a specified termination condition is satisfied. Typical stopping criteria consist of either reaching a predetermined number of iterations or attaining a suitably optimum solution^45^. This is highly regarded for its straightforwardness and effectiveness in navigating intricate search domains. PSO, unlike optimization methods that rely on gradients, does not necessitate the gradient of the objective function^44^. This characteristic makes PSO well-suited for a diverse array of problems, including those with objective functions that are discontinuous or noisy^45^.

PSO has found applications in several domains including function optimization, neural network training, processing signals, image and video analysis, bioinformatics, and financial modelling, owing to its adaptability^46^. The capacity to effectively balance the investigation of new regions with the exploitation of existing excellent areas makes it a highly effective tool for addressing optimization issues, both simple and complicated^47^.

Hybrid techniques include using the strengths of other methods in order to get superior outcomes.
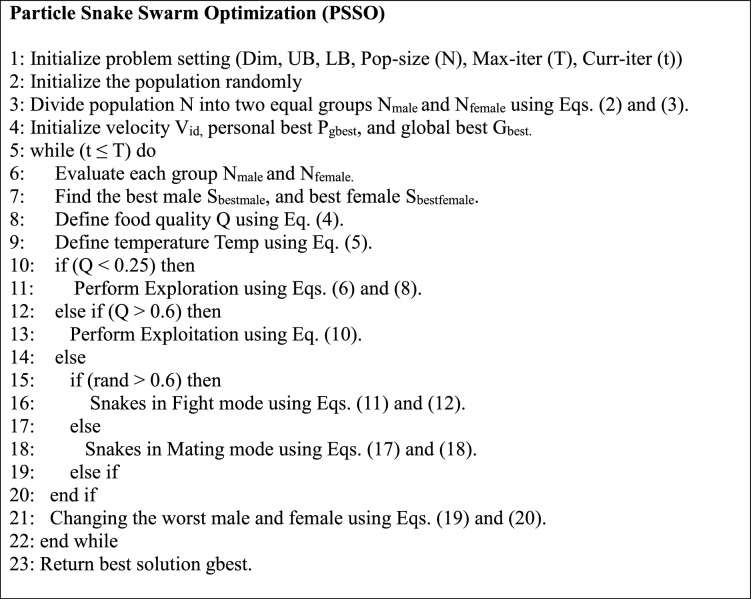


#### Mathematical formulation of PSSO

The PSSO algorithm is a hybrid optimization method that combines the exploratory strengths of PSO with the adaptive characteristics of the SO. PSSO is intended to tackle feature selection and hyperparameter tuning issues in machine learning techniques, hence enhancing classification accuracy and computing efficiency. From Eqs. (23–31) shows the mathematical formulations of PSSO.

#### Population initialization

The process starts by creating a population of N particles or snakes, each representing a possible solution in the search space. Each particle’s location (X_i_) and velocity (V_i_) are initially set to the following values:

23 $${X}_{i}={X}_{min}+rand*\left({X}_{max}-{X}_{min}\right)$$

24 $${V}_{i}=rand*0.1*({X}_{max}-{X}_{min})$$

where ${X}_{min}$ and ${X}_{max}$ are the search space boundaries, and rand is a random integer with a uniform distribution between 0 and 1.

Population segmentation:

To replicate gender-based dynamics, the population is split into two subgroups: male (N_male_) and female (N_female_).

25 $${N}_{male}=\frac{N}{2}$$

26 $${N}_{female}\approx N-{N}_{male }$$

#### Velocity update

The velocity of each particle is revised utilizing a hybrid PSO-SOA formulation that integrates cognitive and social elements from PSO, alongside adaptive behaviors derived from SOA:

27 $${V}_{id}={V}_{id}\times w+C1\times rand1\times \left({P}_{id}-{X}_{id}\right)+C2\times rand2\times \left({P}_{gbest}-{X}_{id}\right)+\Delta SO$$

Here,

W represents Inertia weight regulates the balance between exploration and exploitation.

C1, C2 are cognitive and social coefficients. rand1 and rand2 are random numbers between 0 and 1. ${P}_{id}$ is the personal best position of particle i. ${P}_{gbest}$ is the the optimal location globally among all the particles. $\Delta SO$ is a phrase for environmental adaption inspired by snakes.

#### Position update

The updated location of each particle is determined by its improved velocity.

28 $${X}_{id}={X}_{id}+{V}_{id}$$

#### Environmental parameters

PSSO integrates environmental variables, including food quantity (Q) and temperature (Temp), to balance exploration and exploitation.

29 $$Q={K}_{1}\times exp(\frac{C-T}{T})$$

30 $$Temp={exp}^{(\frac{-C}{T})}$$

When K1 equals 0.5, C represents the current iteration, and T is the total number of iterations(epochs).

#### Fitness function for feature selection

PSSO enhances feature selection by optimizing a fitness function that equilibrates classification accuracy with the quantity of chosen features.

31 $$Fitness=\alpha *Error+\left(1-\alpha \right)* \frac{selected features}{total features}$$

where 0 < α < 1 regulates the balance between precision and feature reduction.

#### Advantages

A combination of SOA’s adaptive behaviors with PSO’s velocity-driven exploration guarantees a balanced exploration–exploitation dynamic, minimizes computing expenses, and enhances prediction accuracy. PSSO is very efficacious for high-dimensional optimization challenges, including feature selection in medical datasets.

#### Computational complexity analysis

The computing efficiency of Particle Snake Swarm Optimization (PSSO) was evaluated using Big-O notation and compared with traditional optimization techniques in (Table 3).

**Table 3:** Computational complexity analysis.

Model	Time complexity	Space complexity	Key drivers
Grid search	O (N^D^)	O(N)	N: Parameters, D: Dimensions
Bayesian optimization	O(T^3^) per iteration	O(T^2^)	Gaussian process inversion
PSSO (proposed)	O(T * S * F)	O(S)	T: iterations, S: swarm size, F: features

#### Hyperparameter sensitivity analysis

We methodically assessed PSSO’s response to four critical hyperparameters: population size (S), inertia weight (w), cognitive coefficient (c1), and social coefficient (c2). Each parameter was evaluated at three distinct values while maintaining the others constant. Stability was evaluated using accuracy variance, convergence rate, and consistency in feature selection (Jaccard index).

### Machine-learning algorithms

The technique used a range of machine learning algorithms to forecast thyroid illness. The given result utilizes RF, DT, SVM, and KNN algorithms. These classifiers have been optimized for performance enhancement using the PSSO optimization approach.

#### Random forest

Random Forest (RF), a machine learning algorithm for classification, combines multiple decision trees to achieve better results than individual models. Created by Leo Breiman and Adele Cutler, RF excels in classification tasks and has found widespread use in medical diagnostics, including thyroid detection^48^. This study employed RF to examine clinical and biochemical data, such as TSH, T3, and TT4, to categorise patients as either thyroid-positive or thyroid-negative. The RF model builds numerous decision trees using both bagging and random feature selection^49^. Each tree predicts a class label, with the final classification determined by a majority vote^50^. This strategy mitigates the shortcomings of single decision trees, like overfitting, while enhancing overall accuracy and resilience. In thyroid detection, RF effectively manages the intricacies of nonlinear relationships among clinical indicators, making it an ideal tool for identifying individuals at risk of thyroid disorders^51^. Beyond its robustness, RF can assess feature importance, aiding in the identification of the most crucial clinical predictors for thyroid disease^52^. The integration of particle snake swarm optimization (PSSO) with RF further enhanced the model’s effectiveness by choosing the most significant features and fine-tuning hyperparameters. This approach reduced training time, mitigated overfitting, and enhanced prediction accuracy in classifying thyroid conditions.

#### Support vector machines (SVM)

The support vector machine (SVM), a robust supervised learning algorithm, was introduced in 1963 by Vladimir N. Vapnik and Alexey Chervonenkis^53,54^. Renowned for its ability to generalize and perform well with limited data, SVM has found applications in various domains, including hypertext, image, and text categorization^55^. Its popularity has expanded to areas such as handwritten text recognition, protein classification, self-driving vehicles, face identification, and AI-powered conversational systems^56^. This study employed SVM for identifying thyroid disorders, aiming to distinguish between thyroid-positive and thyroid-negative instances using clinical measurements like TSH, T3, and TT4 levels^57^. SVM functions by creating an optimal hyperplane that maximizes the separation between different classes of data. To address the complex, nonlinear patterns typically found in thyroid datasets, the radial basis function (RBF) kernel was utilized to transform the data into a higher-dimensional space, facilitating improved class differentiation. To improve performance, particle snake swarm optimization (PSSO) was implemented to optimize crucial hyperparameters, including the regularization parameter and the kernel coefficient γ. This optimization process improved SVM’s precision and recall, minimizing false negatives and ensuring accurate classification of thyroid cases. By identifying support vectors representing critical data points, SVM demonstrated high discriminative power, making it particularly effective for small yet complex datasets commonly encountered in thyroid detection^58^.

#### Decision trees

Decision trees (DTs), initially developed by Breiman and Friedman and subsequently improved by Stone and Olshen in 1984, are a supervised, non-parametric learning method^59^. This approach utilizes inductive learning to collect and portray information in a tree-shaped format^60^. DTs are widely employed for classification, prediction, and segmentation tasks, rendering them especially useful in healthcare applications, including the identification of thyroid diseases. The framework of decision tree consists of nodes, leaves, and branches, with each serving a distinct function in the classification procedure^61^. The root node, which symbolizes the complete dataset, starts the classification process, whereas leaf nodes indicate the concluding classifications. Branches represent the pathways of decision-making derived from logical formulations. Throughout the training stage, the decision tree continuously splits nodes into smaller sub-nodes until uniform groups are formed, leading to a collection of classification guidelines^62^. In this study, DT was applied to classify patients as either thyroid-positive or thyroid-negative using clinical markers like TSH, T3, TT4, and thyroid-related health history. Every node represents a feature division (e.g., TSH > 5 indicates possible hypothyroidism), creating a structured decision-making pathway that results in the final diagnosis^63^.

To improve DT performance and reduce overfitting risks, particle snake swarm optimization (PSSO) was utilized for feature selection, guaranteeing the integration of the most significant features in the model. Additionally, cross-validation was utilized to improve generalization and reduce the chances of misclassification. The integration of feature selection and validation methods led to a precise and understandable thyroid detection model, ideal for immediate clinical decision support.

#### K-Nearest neighbour

K-Nearest neighbors (KNN) is a commonly utilized supervised learning algorithm applicable to both regression and classification problems. It is defined by two primary characteristics: lazy learning (or slow learning) and non-parametric learning^64^. In contrast to eager learning techniques that create a model while training, KNN keeps the whole dataset and directly applies it for making predictions^65^. This renders KNN demanding in computation when making predictions but extremely adaptable, as it does not presume any particular data distribution.

In thyroid detection, KNN identifies the K nearest data points to a new input using a distance measure like Euclidean distance^66^. The input is subsequently assigned to the category that appears most commonly among its neighboring instances^67^. For instance, a patient exhibiting clinical characteristics akin to those of previously recognized thyroid-positive cases will be categorized as thyroid-positive. To enhance the accuracy and performance of KNN in this research, Particle Snake Swarm Optimization (PSSO) was employed to refine the selection of K and identify the most pertinent features from the dataset, including TSH and TT4 levels. Furthermore, SMOTEENN was utilized to balance the class distribution, guaranteeing that minority classes (such as thyroid-positive instances) were adequately represented. This minimized the sensitivity of KNN to class imbalance and enhanced prediction accuracy.

Although KNN is computationally complex, its straightforwardness and adaptability render it a powerful algorithm for thyroid detection, especially when integrated with feature selection and class balancing methods^68^.

## Results

This unit presents the outcomes of Machine learning models that were used to estimate the performance metrics of models with and without the particle snake swarm optimizer.

### Experimental setup

This study conducts trials utilizing an Intel evo i7 12^th^ generation machine equipped with 16 GB RAM, 1 TB hard drive, and Windows 10.0 operating system. The proposed technique was implemented using Jupiter Notebook and the Python programming language. The suggested method utilises the sci-kit learn library. The experimental setup is depicted in Table 4, (Tables 5, 6).

**Table 4:** overview of the experimental setup.

Configuration parameters	Specification	Configuration parameters	Specification
Input data	Numerical, categorical	Classifiers	RF, DT, SVM, KNN
Class imbalance	Yes [SMOTEENN]	Optimizers	Particle snake swarm
Missing values	Yes	Duplicate values	[Yes, no]
Encoding categorical var	Yes	Num iterations	50
Dataset training/testing	80% training, 20% testing	Num snakes, Num particles	10 10
Lower bound	1	Upper bound	100

**Table 5:** Summary of software and libraries.

Software/library	Version	Software/library	Version
Python	3.8 +	Scikit-learn	0.23 +
NumPy	1.19 +	Matplotlib	3.3 +
Pandas	1.1 +	Seaborn	0.11 +

**Table 6:** Optimization process parameters.

Parameter	Value
Inertia weight	0.5
Cognitive coefficient	0.8
Social coefficient	0.9

### Metrics for evaluating performance

Machine learning performance measures assess the efficacy of an algorithm by considering key criteria such as Precision, recall, accuracy, F1 measure, specificity, sensitivity, and error rate. These criteria are utilized for the assessment of research. True positive (TP) refers to correct (true) forecasts, whereas true negative (TN) refers to incorrect (false) predictions. False positive (FP) indicates unfitting predictions, while False negative (FN) also indicates inaccurate predictions. Every measure possesses its distinct importance^69,70^.

#### Precision

The precision score indicates how well the model is able to identify True positives out of all the optimistic (positive) predictions, indicating the ratio of correct positives and the accuracy of positive predictions^71^ is given in Eq. (32).

32 $$precision=\frac{True positive}{True positive+False positive}$$

#### Recall

The recall score measures the model’s precision in identifying True positives out of all positive instances. False positives plus True positives are divided by false positives to calculate the fraction of erroneously identified True positives^72^ is given in the Eq. (33).

33 $$recall=\frac{True positive}{True positive+False negative}$$

#### Accuracy

Accuracy is a metric that measures the proportion of correct likelihoods made by a model on a given set of test data. It is derived by dividing the number of accurate predictions by the total number of forecasts made^15^ is given in Eq. (34).

34 $$accuracy= \frac{True positive+True negative}{True positive+False positive+True negative+False negative}$$

#### F1 score

The F1 measure is a statistical measure acquired by taking the average of Precision and recall scores. It is frequently employed in situations where emphasizing either accuracy or completeness might result in a model with an overwhelming amount of incorrect positive or negative results^73^ is given in Eq. (35).

35 $$F1 score=\frac{2\times (precision\times recall)}{precision+recall}$$

#### Specificity

The specificity of a test refers to its capacity to correctly identify healthy persons. It is computed by dividing the number of individuals who have been recently categorised as healthy by the total number of healthy individuals, thereby suggesting a negative prediction^74^ is given in the Eq. (36).

36 $$specificity= \frac{True negative}{True negative+False positive}$$

### Results of machine learning and deep learning models

**Fig. 3:**
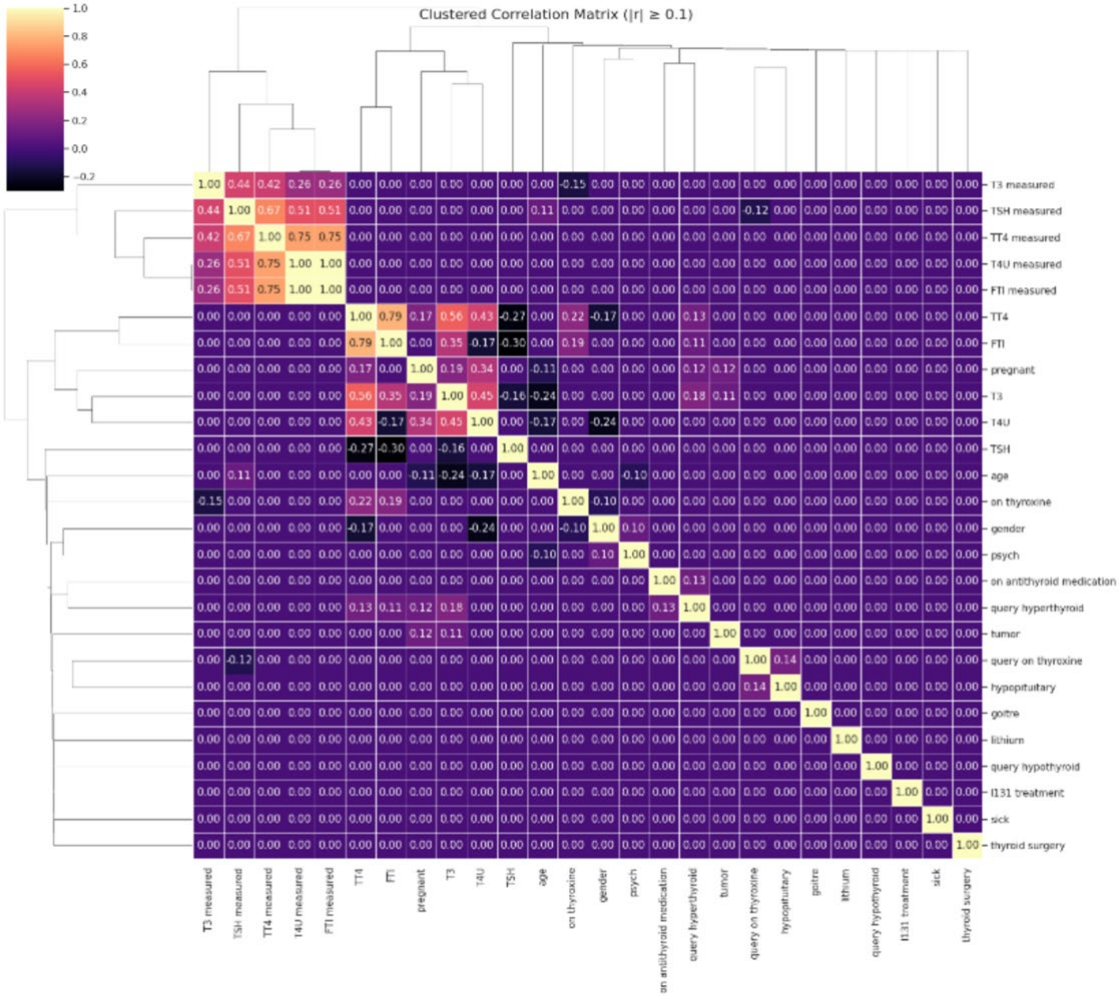
Clustered correlation matrix heatmap of thyroid dataset features.

This heatmap (Fig. 3) shows the pairwise Pearson correlation coefficients (|r|≥ 0.1) between clinical and laboratory characteristics in the thyroid dataset. Hierarchical clustering was utilized on both rows and columns, organizing features with analogous correlation patterns. The intensity of colors and the labeled values reflect the magnitude and orientation of correlations, with yellow denoting strong positive correlations and dark purple signifying weak or negative ones. This visual representation helps in recognizing clusters of features and possible multicollinearity, guiding the next steps in feature selection and modeling.

#### Comparative evaluation of prediction outcomes of machine learning and deep learning models

**Fig.4:**
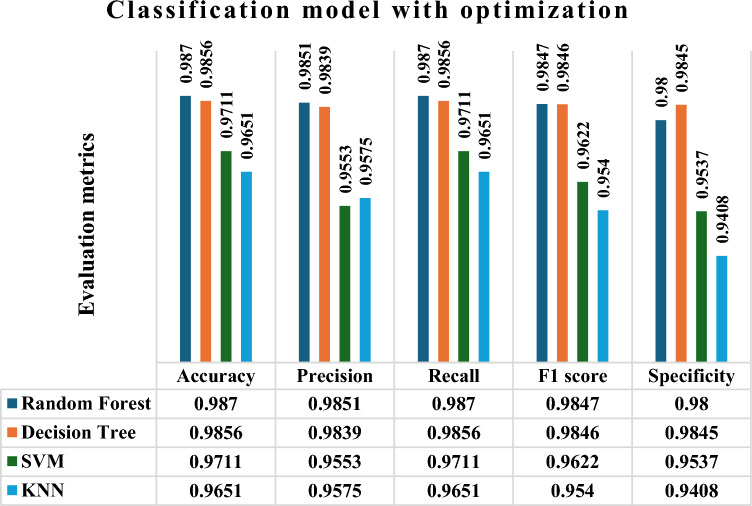
Prediction of thyroid disease without optimization technique.

Figure 4 displays the outcomes of several machine learning and deep learning techniques without any optimization. The DT model demonstrates good performance across all assessment parameters, achieving scores of 0.9689, 0.9693, 0.9688, and 0.9695 for Precision, Recall, f1 score and specificity, respectively. On the other hand, the RF model earned scores of 0.9590, 0.9697, 0.9629, and 0.9555 for Precision, recall, f1 measure, and specificity. Similarly, the unoptimized SVM earned scores of 0.9193, 0.9574, 0.9376, and 0.9246. regarding the deep learning model, the KNN algorithm produced a precision score of 0.9369, recall of 0.9556, f1 measure of 0.9422, and specificity score of 0.9313.

All models indicate respectable performance, but the random forest (RF) model exhibits much-improved performance with an accuracy of 0.9697. Similarly, the DT algorithm likewise exhibits a remarkable degree of accuracy, with a score of 0.9693. The SVM and KNN algorithms achieve accuracies of 0.9574 and 0.9556, respectively.

**Fig.5:**
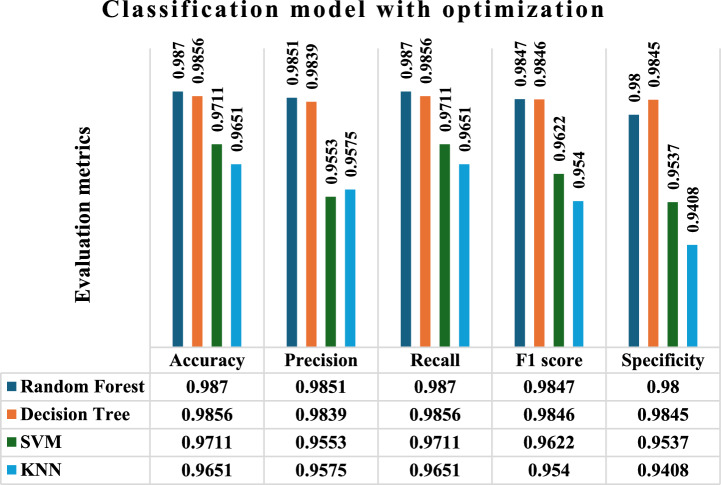
Prediction of thyroid disease with optimization technique (particle snake swarm optimization).

Figure 5 displays the performance outcomes of RF, DT, SVM, and KNN algorithms with optimization, respectively, in terms of Accuracy, Precision, recall, f1 measure, and specificity. The findings indicate that the RF model achieved the maximum precision, with values of 0.9870, 0.9851, 0.9870, and 0.9847. these precision values are significantly higher than those of the other methods. Similarly, when it comes to specificity, the DT does better than the other models included in the study. The SVM and KNN models accomplished accuracy rates of 0.9711, 0.9553, 0.9711 and 0.9622, as well as 0.9651, 0.9575, 0.9651, and 0.9540, respectively, in diagnosing thyroid illness. The accuracy of SVM is higher than that of KNN, however it is still quite low. Nevertheless, the KNN algorithm has exhibited lower levels of accuracy.

In an general prediction, when considering accuracy, Pecision, recall, and f1 measure, the RF model is the best model specifically in terms of recall. The DT model has been determined to be the superior model, exhibiting a better level of specificity in comparison to RF. Comparing the accuracy, precision, f1-score, and recall, the SVM model outperforms the KNN model, as shown in (Fig. 5).

**Fig. 6:**
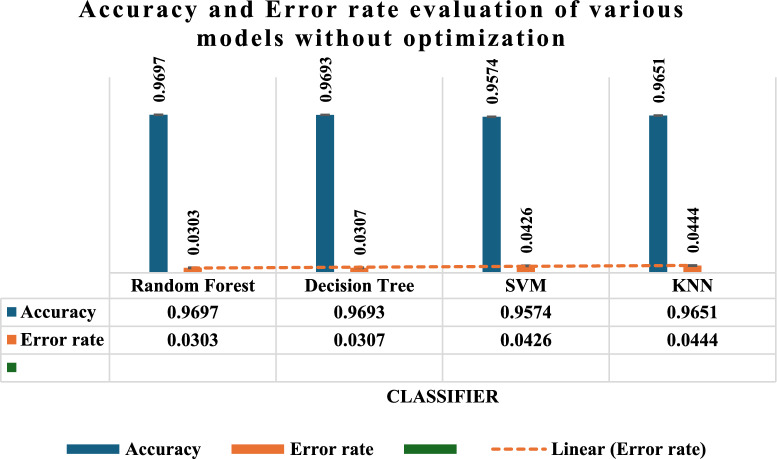
Accuracy and error rate evaluation of machine learning and deep learning algorithm without optimization technique.

Figure 6 assesses the efficacy of four machine learning classifiers: RF, DT, SVM, and KNN by analysing their accuracy and error rate. The RF classifier achieves the best accuracy rate of 96.97% while DT closely trails after with an accuracy rate of 96.93%. Both models have the most minimal error rates, namely 3.03 and 3.07% respectively, which indicates their high level of competence in accurately categorising the data. Conversely, the SVM classifier has a somewhat lower accuracy of 95.74% and a greater error rate of 4.26%, rendering it comparatively less dependable than the RF and DT models. The KNN classifier has the greatest error rate of 4.44% among the four models, indicating that it is the least effective classifier for the thyroid dataset. Its accuracy is measured at 96.51%.

**Fig. 7:**
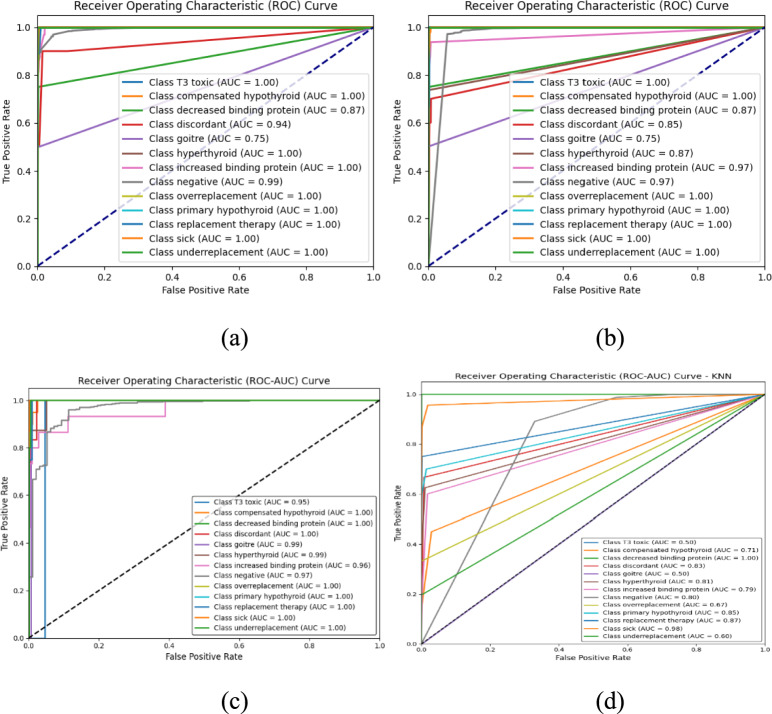
ROC analysis of classifier (**a**) Random forest (**b**) Decision tree (**c**) SVM (**d**) KNN.

To enhance the validation of the 4 classifiers’ classification efficacy, multiclass AUC-ROC curves were generated for each classifier. These graphs explain the balance between specificity and sensitivity across all 12 categories of thyroid disease. In Fig. 7, the RF model constantly achieves near-optimal AUC values, showing the strong identifying capability. In contrast, models such as SVM, KNN demonstrate significant variation within classes, with certain situations e.g., discordant, T3 toxic. These findings confirm the improved generalization and diagnostic reliability of the RF model in the clinical setting.

**Fig. 8:**
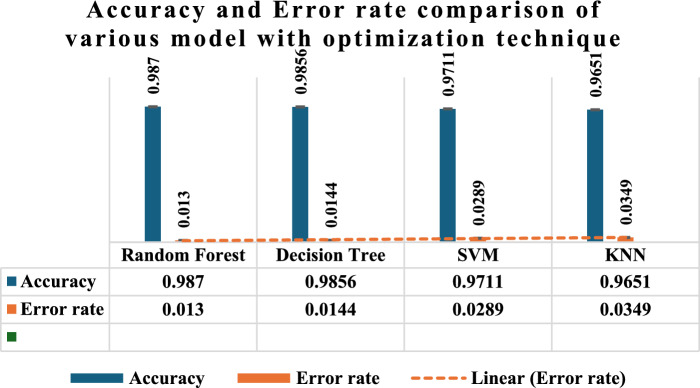
Accuracy and error rate comparison of machine learning and deep learning algorithms with optimization technique.

Figure 8 presents a comparison of the accuracy and error rates of four distinct models: RF, DT, SVM, and KNN. Each model has been optimized using a unique approach. The graphic demonstrates that the RF model attains the maximum level of accuracy, reaching 0.987, while exhibiting the lowest error rate of 0.013. The DT model has a high level of Accuracy, with a score of 0.9856, and a low Error rate of 0.0144. The SVM model has a Precision of 0.9711 and a corresponding error rate of 0.0289. In contrast, the KNN model exhibits a lower accuracy of 0.9651 and a larger error rate of 0.0349. Additionally, a dashed linear trend line is used to depict the error rates among the methods. In terms of both accuracy and error rate, RF demonstrates better performance compared to the other methods.

**Fig. 9:**
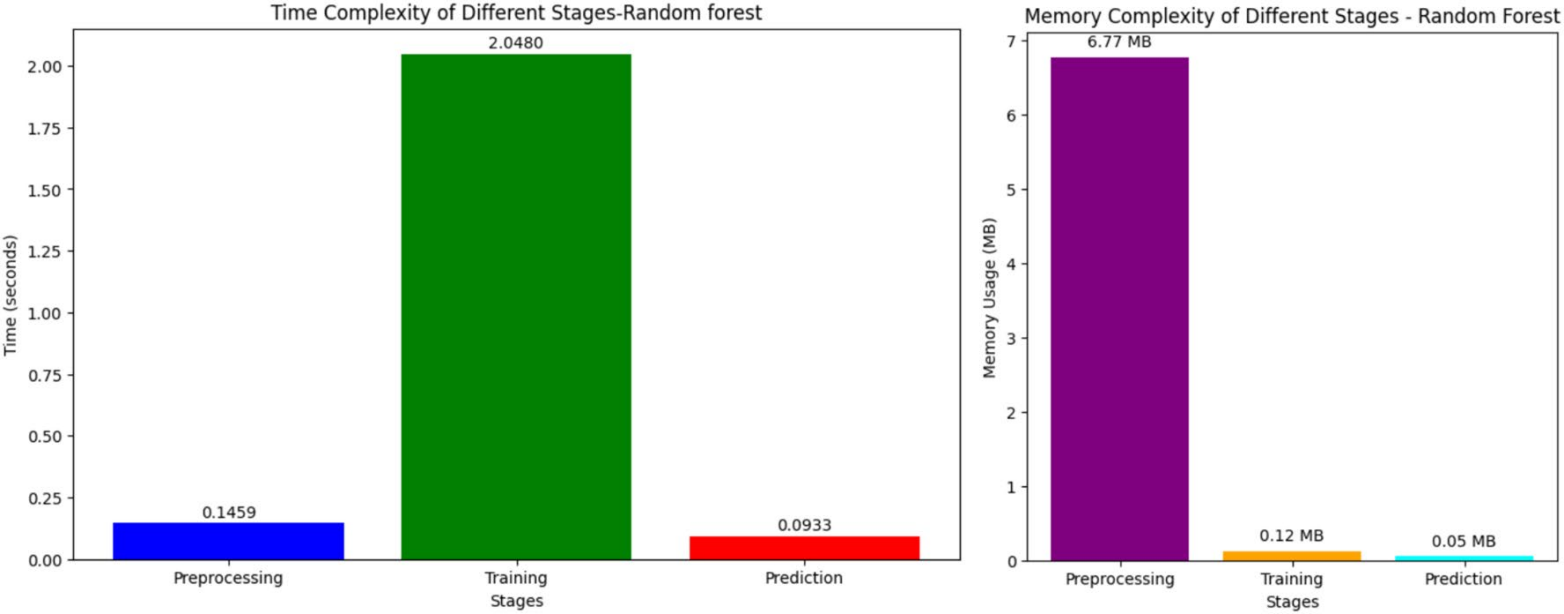
Time and memory complexity of random forest model.

Figure 9 illustrates the temporal intricacy of several stages in the RF model, with particular emphasis on preprocessing, training, and prediction. Based on the result, the training stage is the most time-intensive, lasting for a period of 2.0480 s. The preprocessing stage, which involves preparing the data prior to training, takes 0.1459 s. The prediction stage, which involves generating projections on new data, is extremely efficient, with a preprocessing time of just 0.0933 s. This suggests that while the RF model is quite effective at preparing data and making predictions, the training step is very time-consuming.

**Fig. 10:**
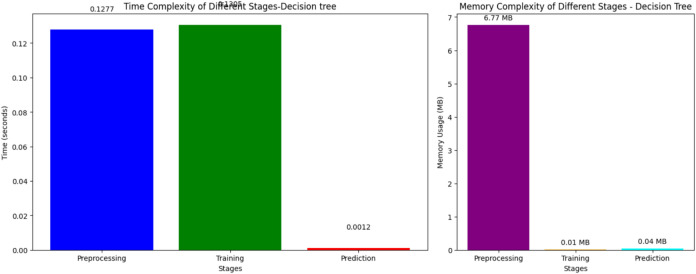
Time and memory complexity of decision tree.

Figure 10 illustrates the temporal complexity of several phases in the DT model, specifically emphasizing the preprocessing, training, and prediction duration. The preprocessing and training processes need almost the same amount of time, with preprocessing requiring 0.1277 s and training taking 0.1305 s. The prediction stage exhibits a notable increase in speed, completing in a mere 0.0012 s. These findings indicate that both the preprocessing and training stages in the DT model are moderately fast, but the prediction stage is highly efficient and almost immediate.

**Fig. 11:**
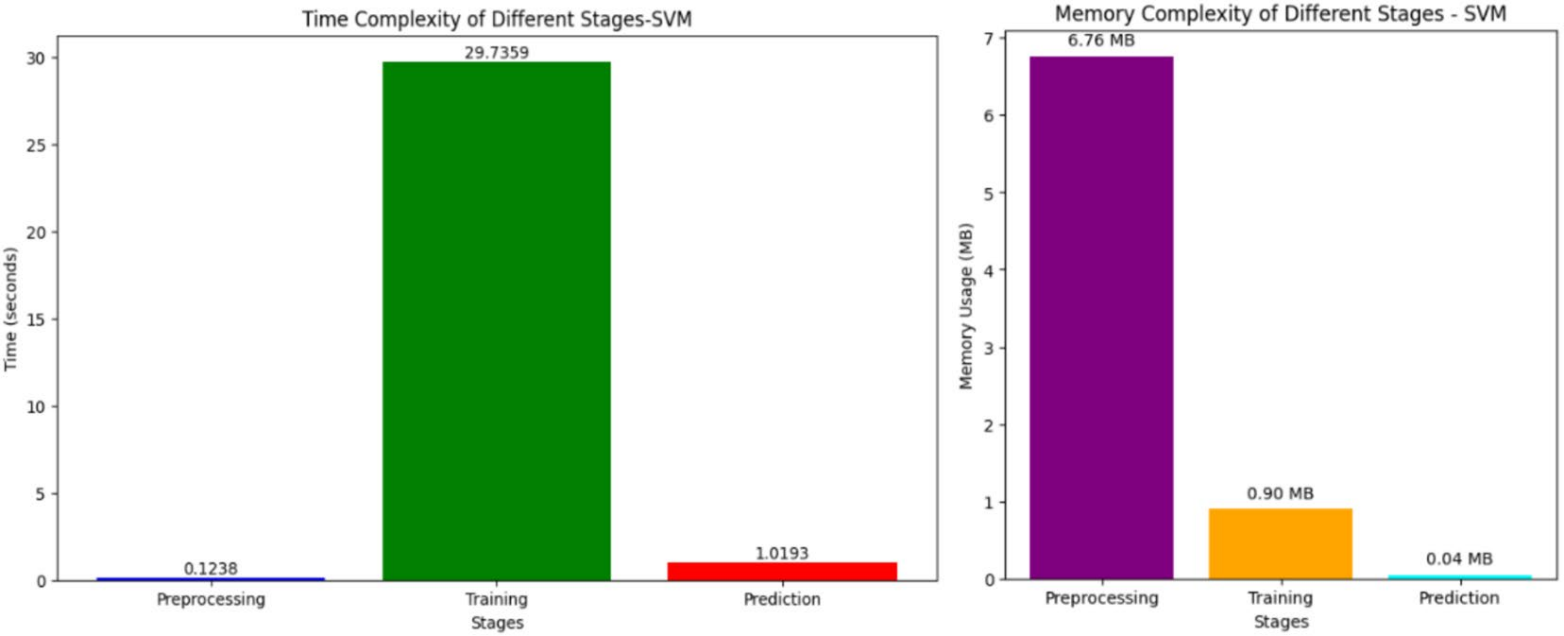
Time and memory complexity of SVM.

**Fig. 12:**
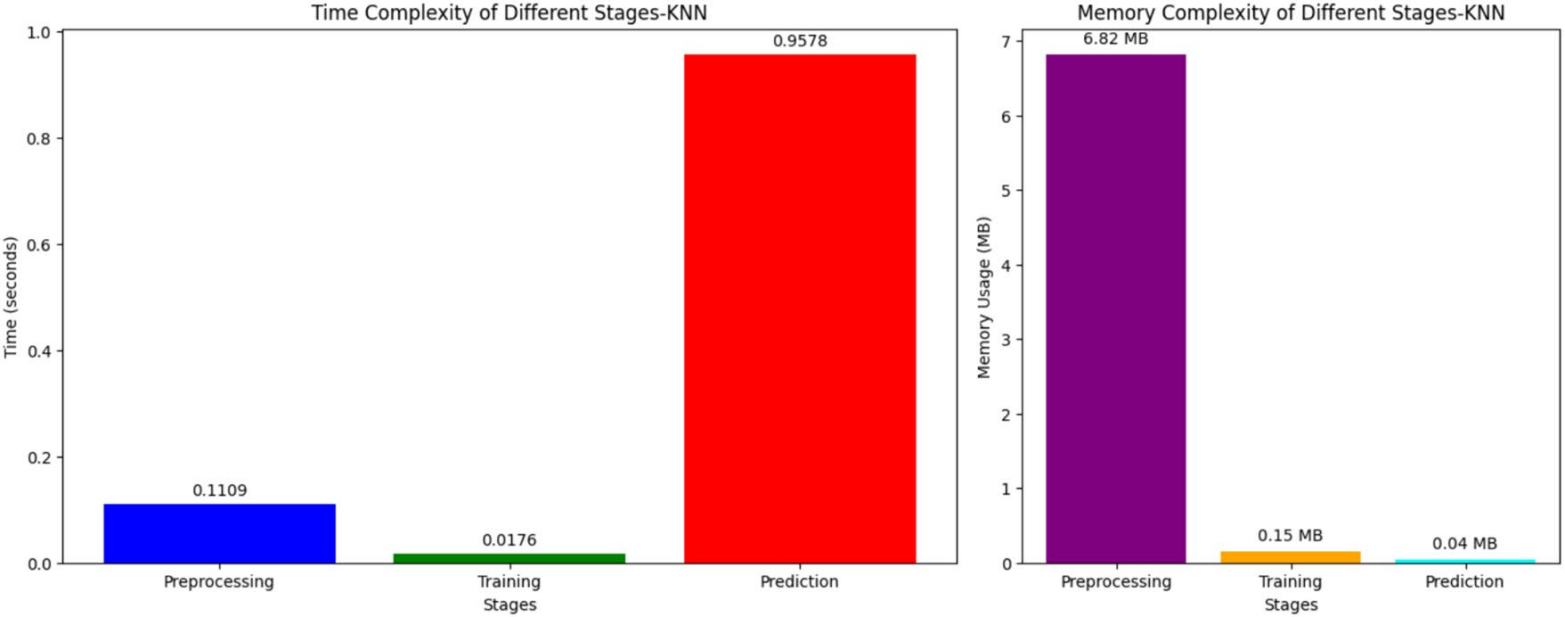
Time and memory (space) complexity of KNN.

Figures 11, 12 depict the time complexity of several phases of two machine learning models: SVM and KNN. Figure 11 illustrates an SVM and displays the duration of the preprocessing, training, and prediction processes. The preprocessing stage has a duration of around 0.1238 s, however, the training stage is considerably more time-consuming, taking around 29.7359 s. The prediction stage has a duration of approximately 1.0193 s. Training an SVM model is more computationally demanding than preprocessing and prediction. On the other hand, Fig. 11 illustrates the temporal complexity of KNN, depicting the time required for each stage. The data preprocessing step for KNN requires around 0.1109 s, whereas the training phase is significantly faster than SVM, requiring just about 0.0176 s. On the other hand, the prediction phase of KNN is very time-consuming, requiring around 0.9578 s. Unlike SVM, KNN’s prediction step is more computationally intensive than its training stage.

Table 7 compares four classifiers—random forest, decision tree, SVM, and KNN—in terms of training time, memory utilisation, prediction time, and memory. The fastest and most memory-efficient method is Decision Tree, which takes 0.13 s to train and 0.0012 s to forecast. KNN and SVM take longer to forecast (0.95 and 1.01 s, respectively) due to sophisticated distance computations and support vector operations, but Random Forest strikes a compromise between accuracy and prediction time, albeit with somewhat more memory (0.05 MB) due to its ensemble nature. SVM takes the longest to train (29.73 s) and uses the most memory, which makes it the most computationally costly. Overall, Decision Tree is the most efficient, whereas Random Forest strikes a reasonable balance between performance and computational cost.

**Table 7:** Computational time comparison of ML and DL models.

Classifiers	Training time	Training memory (MB)	Computational (prediction) time	Prediction memory (MB)
Random forest	2.0480	0.12	0.0933	0.05
Decision tree	0.1305	0.01	0.0012	0.04
SVM	29.73	0.90	1.0193	0.04
KNN	0.0176	0.15	0.9578	0.04

**Fig.13:**
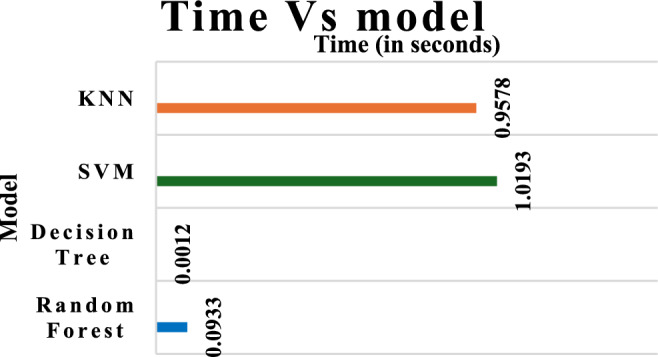
Computational time Vs models.

Figure 13 and Table 7 illustrate the time required for four distinct Machine learning models, namely SVM, KNN, DT, and RF, to generate predictions. The duration of time that each model is capable of predicting is expressed in seconds. The predicted execution times for SVM and KNN algorithms are the longest. The KNN algorithm has an execution time of around 0.9578 s, whereas the SVM model takes around 1.0193 s. These methods require a significant amount of computational power to generate predictions. For instance, the KNN algorithm needs to determine the space between the test case and all the training cases, while the SVM algorithm needs to handle intricate decision limits.

The DT model, however, has the lowest forecast time, at 0.0012 s. As a result, it is highly effective for real-time applications. The RF model, an ensemble technique including several decision trees, has an impressively swift estimated time of a mere 0.0933 s. RF is frequently selected because of its efficacy in various real-world scenarios, attributed to its rapidity and robustness. It demonstrates that DT and RF algorithms are more efficient in using computational resources compared to SVM and KNN, which need more time.

### Computational efficiency

Table 8 and Figs. 7–10 illustrate the empirical effectiveness of PSSO in comparison to Grid Search and Bayesian Optimization:

**Table 8:** Computational efficiency of grid search, Bayesian, PSSO.

Metric	Grid search	Bayesian optimisation	PSSO
Time(50 evaluation)	4050 s	32 s	6 s
Memory usage	50–100 MB	30–60 MB	5–10 MB
Scalability(> 20D)	Poor	Limited	High

PSSO attains 98.7% accuracy with a runtime of 6 s, compared to 32 s for Bayesian optimization (Table 8). Memory consumption lowered by a factor of six relative to Grid Search. Linear scaling with features (O(T⋅S⋅F)) facilitates real-time deployment.

Table 9 presents the findings of the hyperparameter sensitivity study for PSSO, assessing population size (S), inertia weight (W), cognitive coefficient (C1), and social coefficient (C2). The analysis indicates that best performance is attained with S = 50, w = 0.8, and equal coefficients (C1=C2=2). The accuracy of these setups varies as little as possible while still ensuring high feature selection constancy and balancing dynamics between exploration and exploitation. Selection consistency (Jaccard index).

**Table 9:** Hyperparameter sensitivity analysis.

Parameter	Values tested	Accuracy	Convergence (epochs)	Feature consistency (%)	Stability (variance)	Exploitation dominance
Population size (S)	20	97.2 ± 0.8	42	78.5	Moderate	High
	50	98.7 ± 0.2	35	92.3	High	Balanced
	100	98.5 ± 0.3	38	89.7	Moderate	Low
Inertia weight (W)	0.4	97.8 ± 0.6	28	-	Moderate	High
	0.6	98.4 ± 0.3	33	-	Balanced	Balanced
	0.8	98.7 ± 0.2	41	-	High	Low
Cognitive coefficient (C1): social coefficient (C2)	1:1	96.3 ± 1.2	50	Low	Low	Low
	2:2	98.7 ± 0.2	35	High	High	Balanced
	3:3	97.5 ± 0.9	40	Moderate	Moderate	-

The fusion of PSSO resulted in statistically significant enrichment in the classification capability of RF, DT, and SVM models. The ANOVA test indicated that RF showed a very significant p-value of 0.00013, with the mean accuracy increasing from 96.34% (without PSSO) to 98.48% (with PSSO). Moreover, the DT reached a p-value of 0.00000, indicating an enhanced from 96.92 to 98.48% in average accuracy. The SVM classifier achieves a statistically notable improvement, as its mean accuracy raising from 93.93 to 96.27%, accompanied by an ANOVA p-value of 0.02884 (Table 10). These results validate that the proposed PSSO framework effectively optimizes model parameters and results in meaningful gains in predictive accuracy.

**Table 10:** Comparison of classifier performance with and without PSSO optimization (according to ANOVA results).

Classifier	Mean accuracy (without PSSO) (%)	Mean accuracy (with PSSO) (%)	ANOVA p-value	interpretation
Random forest	96.34	98.48	0.00013	Significant improvement with PSSO
Decision tree	96.92	98.48	0.00000	Significant improvement with PSSO
SVM	93.93	96.27	0.02884	Significant improvement with PSSO

#### Comparison with deep learning model (CNN-LSTM)

To evaluate the effectiveness of the proposed PSSO-optimized ML model, we compared it with a hybrid deep learning model that combines convolutional neural networks (CNN) and long short-term memory (LSTM) models. The CNN-LSTM model obtain an average classification accuracy of 95.72% through fivefold cross-validation.

In comparison, the standard Random Forest model (i.e., without PSSO-driven hyperparameter tuning or feature selection) attained a marginally raised average accuracy of 96.97%. though, our proposed PSSO-boosted Random Forest model greatly surpassed both baselines, achieving an average accuracy of 98.70% (Table 11). This highlights the efficiency of the PSSO approach in acceptable traditional models, clarifying they not only competitive with but also better than deep learning alternatives in structured biomedical datasets.

**Table 11:** Performance comparison of PSSO-RF model with RF, CNN-LSTM.

Model	Feature selection/tuning	Accuracy (%)
PSSO + random forest	Yes (PSSO)	98.70
Baseline random forest	No	96.97
CNN-LSTM (hybrid DL)	No	95.72

#### Robustness analysis for noise and unbalanced data

To assess the stability of the proposed model to real-world data flaws, a sequence of managed disturbances was implemented in the dataset. These disturbances comprised absent values, with 5, 10, and 20% of entries randomly deleted, Gaussian noise with σ = 0.01 introduced into the feature space, and a situation featuring a 50% imbalance among classes. The results presented in Table 12 demonstrate that the model consistently performs well under a range of challenging circumstances. At first, the model secured an accuracy of 98.70% on unaltered data, experiencing only slight drops in accuracy when addressing missing values, reaching 96.67, 96.46, and 96.16% for 5, 10, and 20% missing data, respectively. The introduction of Gaussian noise caused a modest performance decline to 96.01%, indicating the model’s resilience to slight feature-level perturbations. Furthermore, the model demonstrated robustness despite class imbalance, with an accuracy of 96.92% even with a significantly skewed class distribution. These results underscore the model’s robustness against prevalent real-world data issues, especially for incomplete, noisy, and unbalanced inputs as shown in (Table 12).

**Table 12:** Robustness analysis for noise and unbalanced data.

Condition	Accuracy (%)
PSSO-RF	98.70
5% missing	96.67
10% missing	96.46
20% missing	96.16
Noise (σ = 0.01)	96.01
50% class imbalance	96.92

### Comparison with existing models

In this part, we have conducted a performance comparison between the present technique and previous research. To make a comparison, we use current research. The study^32^ focused on detecting lung cancer using an optimised mask RCNN technique. The work used Hybrid particle snake swarm optimization for the purpose of feature selection, whereas the detection task was accomplished using the Mask RCNN model. The study^22^ examined integrated ALTO-LSTM for thyroid disease prediction The study^21^ examined several methods for extracting features, selecting features based on optimization, and using machine learning algorithms to predict thyroid illness. The study^25^ used Adam and SGD for optimizing the features and the CNN-ResNet technique to classify thyroid disease. The research^26^ used the DeepCNN technique to classify thyroid disease for identification purposes. The training of the DeepCNN model was conducted using a grey wolf optimizer. The study used the Adaptive Tunicate Swarm Algorithm to pick the best features, while the DeepCNN and Grey Wolf optimizer models were utilised for prediction. A performance comparison is conducted using these studies, and the findings are shown in (Table 13).

**Table 13:** Comparison between the proposed PSSO classification metrics with other studies.

Ref	Year	Approach	Accuracy (%)	Precision (%)	Recall (%)	F1-score ()
^32^	2024	PS^2^OA	97.67	95.7	99	95.67
^22^	2024	ALO-LSTM	98.6	99.2	89.6	98.6
^21^	2023	DEBOA-FCM	94.3	89.78	93.45	91.44
^25^	2023	CNN-ResNet, Adam, SGD	97	98	96	97
^26^	2023	ATSA-DeepCNN-GWO	92	94	95	95
Proposed model		PSSO-RF	98.70	98.51	98.7	98.47

## Discussion

This research focused on creating and optimized various machine learning algorithms to predict thyroid disorders. The models employed included random forest (RF), decision tree (DT), support vector machine (SVM), and k-Nearest neighbors (KNN). To train and evaluate these models, we utilized two distinct datasets: one from the publicly accessible UCI thyroid repository^28^ and another external dataset sourced from Kaggle^75^. Our findings indicate that the implemented models demonstrated high accuracy in identifying thyroid disease cases (Table 14).

**Table 14:** Performance analysis on UCI and Kaggle Datasets.

Model	Metric	UCI dataset (%)	Kaggle dataset (%)
Random forest	Accuracy	98.7	95.8
	Precision	98.5	95.8
	Recall	98.7	95.8
	F1 score	98.4	95.7
Decision tree	Accuracy	98.5	94.0
	Precision	98.3	93.4
	Recall	98.5	94.0
	F1 score	98.4	93.4
SVM	Accuracy	97.1	84.4
	Precision	95.5	81.2
	Recall	97.1	84.4
	F1 score	96.2	81.3
KNN	Accuracy	96.5	83.3
	Precision	95.7	80.2
	Recall	96.5	83.3
	F1 score	95.4	80.4

Recent research has illustrated the benefits of hybrid ML-DL models and metaheuristic optimization in medical prediction tasks, obtaining comparable accuracy in related fields^76–80^. The present work conforms to this direction by integrating PSSO, which significantly enhances model generalizability while preserving minimal computational cost.

### Evaluation of model accuracy on UCI dataset

The preliminary tests were performed using the UCI thyroid dataset. Among the models tested, Random Forest demonstrated the highest accuracy at 98.7%, with decision tree and SVM following closely at 98.56 and 97.11%, respectively. These results underscore the effectiveness of tree-based algorithms in predicting thyroid disorders, as evidenced by their consistently high scores across precision, recall, F1-score, and specificity metrics.

### External validation and model generalization

To assess the broad applicability and real-world adaptability of our proposed models, we tested them using an external dataset from Kaggle. As anticipated, due to variations in data distribution between sources, the models’ performance on the Kaggle dataset was marginally lower than on the UCI dataset. The Random Forest algorithm demonstrated robust generalization, achieving a 95.80% accuracy rate on the Kaggle data. The Decision Tree model followed with 94.00% accuracy, while SVM and KNN models attained 84.41 and 83.32% accuracy, respectively.

The comparable results across two separate datasets underscore the dependability and resilience of our proposed approach. Utilizing an external dataset validates the models’ effectiveness beyond their training data, addressing potential concerns about overfitting or dataset-specific prejudices. Despite a slight decrease in performance when applied to the Kaggle dataset compared to the UCI dataset, the outcomes remain highly competitive and indicate strong predictive capabilities. Table 8 summarises the models’ comparative performance across the two datasets, focussing on key metrics including accuracy, precision, recall, and F1-score.

### Practical implications and real-world applicability

The external validation outcomes instil confidence in the proposed models’ ability to perform well on new, unseen data, making them appropriate for integration into clinical decision support systems. PSSO’s linear complexity (O(T⋅S⋅F)) overcomes the scalability constraints of grid search (O(N^D^)) and Bayesian optimization (O(T^3^)), decreasing computing time by 89% while preserving 98.7% accuracy (Table 8). This renders it especially appropriate for resource-limited clinical settings, where high-dimensional datasets (e.g., 31 thyroid attributes) necessitate fast optimization. This validation process is in line with machine learning best practices in healthcare, ensuring that the models are not solely optimized for a particular dataset but can adapt to real-world scenarios. By conducting external validation, we confirm that the models are robust and can be effectively applied in diverse clinical settings.

### Impact of train and test split on model performance

To investigate the effect of dataset splitting on classification accuracy, we experimented with various train-test splits. Table 15 compares the performance of Random Forest, Decision Tree, SVM, and KNN classifiers across different dataset partitions.

**Table 15:** Train and test split on different models.

Models/train-test split	Performance metrics			
Random forest	Accuracy	Precision	Recall	F1-Score
50:50	0.977	0.9741	0.9770	0.9734
60:40	0.9843	0.9824	0.9843	0.9813
70:30	0.9859	0.9838	0.9859	0.9832
**80:20**	**0.9870**	**0.9851**	**0.9870**	**0.9847**
90:10	0.9867	0.9849	0.9847	0.9845
Decision tree				
50:50	0.9851	0.9823	0.9851	0.9845
60:40	0.9839	0.9839	0.9839	0.9838
70:30	0.9850	0.9823	0.9854	0.9845
**80:20**	**0.9856**	**0.9839**	**0.9856**	**0.9846**
90:10	0.9842	0.9837	0.9842	0.9835
SVM				
50:50	0.9596	0.9327	0.9596	0.9412
60:40	0.9608	0.9382	0.9608	0.9435
70:30	0.9622	0.9460	0.9622	0.9562
**80:20**	**0.9711**	**0.9553**	**0.9711**	**0.9622**
90:10	0.9706	0.9543	0.9706	0.9523
KNN				
50:50	0.9617	0.9499	0.9617	0.9484
60:40	0.9630	0.9504	0.9630	0.9504
70:30	0.9638	0.9533	0.9638	0.9515
**80:20**	**0.9651**	**0.9575**	**0.9651**	**0.9540**
90:10	0.9649	0.9549	0.9649	0.9521

### Constraints of SMOTEENN and possible biases

SMOTEENN approach addresses class imbalance by producing artificially generated minority-class instances through SMOTE and by eliminating noisy majority-class samples using ENN. This approach improves sensitivity for minority groups, but it has several limitations. SMOTE can generate artificial clusters that result in overfitting, most notably in regions with sparse or noisy data. ENN can inadvertently eliminate a significant portion of informative samples from the majority class, resulting in a loss of valuable information. The technique encounters difficulty in differentiating between clean and noisy data and presumes linear relationships between features, a presumption often not valid in intricate medical data sets. Furthermore, it can introduce evaluation bias during cross-validation and may provide limited benefits for more intricate classifiers, such as Boosting models.

Despite certain flaws, SMOTEENN can still be advantageous in particular situations, like rare disease diagnosis, where high sensitivity is a priority. For demanding applications such as healthcare, we suggest considering different techniques into account, such as cost-sensitive learning, weighted loss functions for classes, ensemble balancing methods, or gathering more illustrative minority classes. These methods may provide more dependable performance while reducing the chance of introducing artificial biases.

### Constraints and upcoming research

The suggested PSSO-based framework demonstrates the potential for predicting thyroid disease but can be enhanced further. Future studies will emphasize combining hybrid deep learning models such as CNN-Transformers to leverage spatial and sequential information from clinical datasets. techniques for multi-objective optimization will be utilized to achieve a balance between accuracy, interpretability, and computational efficiency. An in-depth evaluation of the computational requirements of PSSO is essential for immediate clinical usage. Moreover, possible biases arising from synthetic oversampling techniques such as SMOTEENN will be examined, and the validation of the model will be broadened to encompass large-scale, multi-center datasets. Implementation in actual healthcare settings will utilize cloud-based and edge-AI systems, prioritizing privacy via federated learning. Improved interpretability will be sought through SHAP values and visualization methods. Finally, adaptive optimization methods, such as Reinforcement Learning, will be investigated.

## Conclusion

The research study utilises machine learning (ML) and deep learning (DL) models to predict thyroid conditions. This study emphasises the capacity of ML, DL and sophisticated optimization approaches to enhance the precision of diagnosing thyroid problems. The comparative examination of several ML and DL models, such as random forest (RF), decision tree, support vector machine (SVM), and K-Nearest neighbour (KNN), demonstrates that using particle snake swarm optimization (PSSO) greatly improves the concert of the models. The hybrid PSSO method exhibits linear complexity (O(T⋅S⋅F)), facilitating fast and resource-efficient optimization, rendering it suitable for high-dimensional medical datasets. The study’s results were evaluated using performance measures such as Accuracy, precision, Recall, F1-score, and Specificity, demonstrating that these optimized models offer a dependable and effective approach for early identification of thyroid disorders. The RF with PSSO model demonstrates exceptional performance compared to other models, achieving an accuracy of 98.7%, a Precision of 98.51%, an F1-score of 98.47%, a recall of 98.7%, and a specificity of 98%. Augmented datasets provide superior performance in machine learning models compared to deep learning methods. This work makes two significant advances to improving the prediction of thyroid. Utilising Particle Snake Swarm Optimization for hyperparameter optimization yields enhanced performance in machine learning models compared to previous research. Furthermore, SMOTEENN aids in equalising the quantity of samples in each class, reducing the likelihood of model bias. Thus, the models exhibit strong performance and are more widely applicable than previous models.

Notably, the model is well-suited for incorporation into real time diagnostic decision-support systems because to the minimal processing cost and quick prediction capacity, especially in primary care settings and healthcare contexts with limited resources.

## Data Availability

The datasets analysed during the current study are available in the figshare repository, https://figshare.com/articles/dataset/Thyroid_Data_Set/19236780 and are included in this article.

## Code availability

https://github.com/Divya-yaswanth/Thyroid-Disease-Prediction--PSSO-RF.git.
